# Adipsin-Dependent Secretion of Hepatocyte Growth Factor Regulates the Adipocyte-Cancer Stem Cell Interaction

**DOI:** 10.3390/cancers13164238

**Published:** 2021-08-23

**Authors:** Masahiro Mizuno, Behnoush Khaledian, Masao Maeda, Takanori Hayashi, Seiya Mizuno, Eiji Munetsuna, Takashi Watanabe, Seishi Kono, Seiji Okada, Motoshi Suzuki, Shintaro Takao, Hironobu Minami, Naoya Asai, Fumihiro Sugiyama, Satoru Takahashi, Yohei Shimono

**Affiliations:** 1 Department of Biochemistry, Fujita Health University School of Medicine, Toyoake 4701192, Japan or 81017035@fujita-hu.ac.jp (M.M.); behnoush.khaledian@fujita-hu.ac.jp (B.K.); masao.maeda@fujita-hu.ac.jp (M.M.); thayashi@fujita-hu.ac.jp (T.H.); mntneiji@fujita-hu.ac.jp (E.M.); t-watana@fujita-hu.ac.jp (T.W.); 2 Department of Pathology, Fujita Health University School of Medicine, Toyoake 4701192, Japan; naoya.asai@fujita-hu.ac.jp; 3 Laboratory Animal Resource Center, Transborder Medical Research Center, Faculty of Medicine, University of Tsukuba, Tsukuba 3058575, Japan; konezumi@md.tsukuba.ac.jp (S.M.); bunbun@md.tsukuba.ac.jp (F.S.); satoruta@md.tsukuba.ac.jp (S.T.); 4 Division of Breast and Endocrine Surgery, Kobe University Graduate School of Medicine, Kobe 6500017, Japan; kono_s@me.com (S.K.); s.takao@hp.pref.hyogo.jp (S.T.); 5 Division of Hematopoiesis, Joint Research Center for Human Retrovirus Infection, Kumamoto University, Kumamoto 8600811, Japan; okadas@kumamoto-u.ac.jp; 6 Department of Molecular Oncology, Fujita Health University School of Medicine, Toyoake 4701192, Japan; motosuzu@fujita-hu.ac.jp; 7 Division of Medical Oncology/Hematology, Kobe University Graduate School of Medicine, Kobe 6500017, Japan; hminami@med.kobe-u.ac.jp

**Keywords:** adipocyte, cancer stem cells, adipsin, hepatocyte growth factor, breast cancer

## Abstract

**Simple Summary:**

Obesity, which is characterized by the excess of adipose tissue, is associated with an increased risk of multiple cancers. We have previously reported that adipsin, a secreted factor from adipocytes, enhances cancer cell proliferation and stem cell properties. In this study, we found that adipsin affected adipocytes themselves and enhanced their secretion of hepatocyte growth factor (HGF). We found that HGF enhanced the adipocyte-cancer cell interactions as a downstream effector of adipsin. Understanding the adipocyte-cancer cell interaction will provide a novel strategy to treat cancers whose initiation, invasion, and metastatic progression are associated with adipose tissues.

**Abstract:**

Adipose tissue is a component of the tumor microenvironment and is involved in tumor progression. We have previously shown that adipokine adipsin (CFD) functions as an enhancer of tumor proliferation and cancer stem cell (CSC) properties in breast cancers. We established the Cfd-knockout (KO) mice and the mammary adipose tissue-derived stem cells (mADSCs) from them. Cfd-KO in mADSCs significantly reduced their ability to enhance tumorsphere formation of breast cancer patient-derived xenograft (PDX) cells, which was restored by the addition of Cfd in the culture medium. Hepatocyte growth factor (HGF) was expressed and secreted from mADSCs in a Cfd-dependent manner. HGF rescued the reduced ability of Cfd-KO mADSCs to promote tumorsphere formation in vitro and tumor formation in vivo by breast cancer PDX cells. These results suggest that HGF is a downstream effector of Cfd in mADSCs that enhances the CSC properties in breast cancers.

## 1. Introduction

Cancer stem cells (CSCs) are the cell sub-population of a specific tumor, which are characterized by both a high tumorigenic capacity upon serial transplantation and an ability to generate tumors that recreate the cellular diversity of the parent lesions [1,2]. Breast cancer is the first solid tumor in which the existence of the cells with CSC properties—enriched in a CD44^+^/CD24^−/low^ population in breast cancers—is experimentally proposed [3]. We and others have identified that the stem cell properties of both breast CSC and normal mammary stem cells are epigenetically regulated by microRNAs, including *miR-200c* and *miR-142*, which suppress the expression of a stem cell gene *BMI1*, and *APC*, a suppressor of WNT signaling pathway, respectively [1,4,5]. Furthermore, factors and pathways such as ZEB1, ZEB2, Sox2, Sox9, Wnt pathway, hedgehog pathway, hippo pathway, and notch pathway are associated with CSC properties in breast cancers [1,2]. CSCs are responsible for tumor progression and therapy resistance. We have recently reported that metastatic progression of breast CSC is characterized by the upregulation of S100A10 and the downregulation of miR-93 [6,7].

Interaction of CSCs with their specific microenvironments, known as stem cell niches, is critical for the maintenance of stem cell properties [8]. In the case of mammary tissue, adipose tissue is an indispensable niche for normal mammary stem cells to develop the mammary epithelium [9,10], and for breast CSCs to develop cancers [3]. We have previously shown that adipsin (complement factor D, CFD), an adipokine secreted predominantly from adipocytes, enhances proliferation and CSC properties of breast cancer patient-derived xenograft (PDX) cells; and this effect is mediated by C3a, a downstream product of adipsin, produced in the alternative pathway of the complement system [11].

Adipsin is first identified as a gene that showed differentiation-dependent expression in cultured mouse adipocyte cell lines and then proved to be identical to complement factor D (CFD) [12]. Whereas the majority of complement proteins are produced in the liver, adipsin is mostly produced in adipose tissues and is also expressed in monocytes and macrophages [12]. In the presence of complement factors D and B, spontaneous hydrolysis of C3 results in the formation of C3 convertase that cleave C3 to produce C3a and C3b in the alternative pathway of the complement system [13]. C3b reacts in the complement pathway to produce membrane-associated complex for the lysis of pathogenic cells, and an anaphylatoxin C3a binds to its own receptors to activate immune responses. Furthermore, in addition to the roles in inflammation and tumor immunity [14,15], adipsin is involved in various biological mechanisms, such as adipocyte differentiation, adipose tissue expansion in bone marrow and liver, and insulin secretion from β cells in diabetes [16,17,18,19,20].

In this study, we established the Cfd-knockout mice and the mammary adipose tissue-derived stem cells (mADSCs) from their mammary fat pad. We found that Cfd-KO mADSCs significantly reduced their ability to enhance tumorsphere formation that reflected CSC properties of breast cancer PDX cells. We then found that the secretion of HGF from mADSCs was Cfd-dependent, and HGF effectively alleviated the suppressive effect of Cfd-KO on tumorsphere formation. These results suggest that Cfd-dependent HGF secretion from mADSCs is one of the molecular mechanisms for ADSC-CSC interaction in breast cancer.

## 2. Materials and Methods

### 2.1. Ethics Statements

Human primary breast cancers were obtained from patients admitted to the Division of Breast and Endocrine Surgery of Kobe University Hospital. The research was pre-approved by Kobe University’s Institutional Review Board (permission number: 1299 and 1481) and Fujita Health University’s Institutional Review Board (permission number: HM19-105) and was conducted in accordance with recognized ethical guidelines. All patients included in the study provided written informed consent. Animal experiments were performed with the approval of Fujita Health University’s Animal Care and Use Committee (permission number: AP19062) and carried out according to the Animal Experiment Regulations of Fujita Health University.

### 2.2. Generation of Cfd-Knockout (KO) Mouse

**Figure 1:**
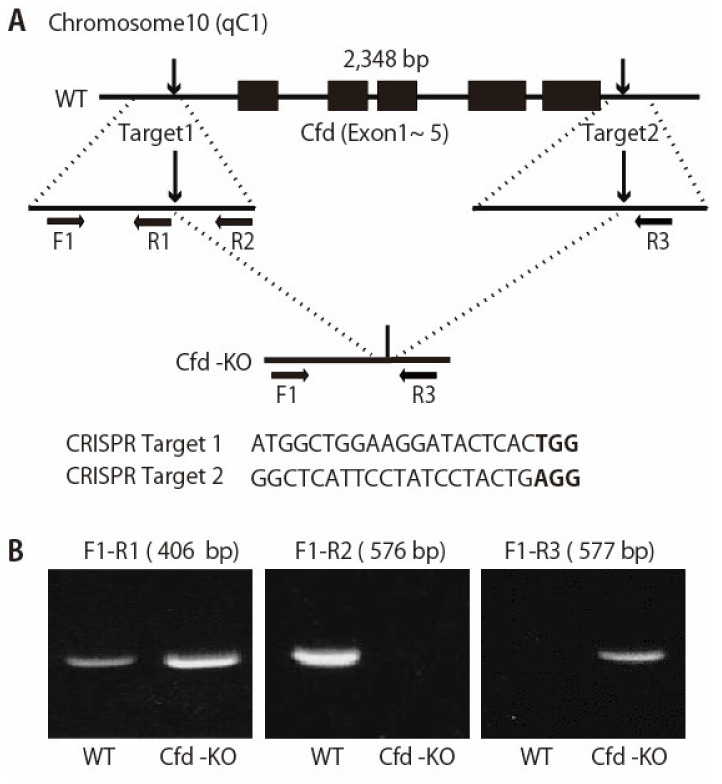
Generation of Cfd-null mice. (**A**) Schematic representation of the *Cfd* locus in the mouse genome. *Cfd* gene is located on chromosome 10. Two sgRNAs were designed which targeted the upstream and downstream of the full coding sequence of the *Cfd* gene. PAM sequences are marked in bold. F1, R1, R2, and R3 represent the position of the primers used to genotype wild type (WT) and KO alleles. (**B**) PCR analysis of the genomic DNA obtained from the mouse tail at 3 weeks of age. The band for WT allele (primers used: F1 and R2, 576 bp) and that for KO allele (primers used: F1 and R3, 577 bp) were determined. Deletion of the Cfd sequence was confirmed by sequencing.

Guide RNA (gRNA): the guide RNA (gRNA) target sites were selected for mouse *Cfd*. The gRNA 1 and gRNA 2 sites were chosen to delete the full coding sequence of *Cfd* (Figure 1A). The gRNAs were synthesized and purified by GeneArt Precision gRNA Synthesis Kit (Thermo Fisher Scientific, Waltham, MA, USA) and dissolved in Opti-MEM (Thermo Fisher Scientific, Waltham, MA, USA).

The pregnant mare serum gonadotropin (PMSG) (5 units) and the human chorionic gonadotropin (hCG) (5 units) were intraperitoneally injected into sexually mature female C57 BL/6 J mice (Charles River Laboratories, Kanagawa, Japan) with a 48-h interval, and unfertilized oocytes were collected from their oviducts. We then performed in vitro fertilization with these oocytes and sperm from sexually mature male C57 BL/6 J mice (Charles River Laboratories, Kanagawa, Japan) according to standard protocols. Five hours later, the gRNAs (25 ng/μL each) and GeneArt Platinum Cas9 Nuclease (Thermo Fisher Scientific, Waltham, MA, USA), (100 ng/μL) were electroplated to zygotes by using NEPA 21 electroplater (NEPAGNENE, Chiba, Japan) in the same condition that we have reported [21]. After electroporation, fertilized eggs that had developed to the two-cell stage were transferred into oviducts in pseudo-pregnant ICR females, and newborns were obtained.

The founders were genotyped by PCR followed by DNA sequencing analysis. The positive founders were bred to the next generation (F1) and subsequently genotyped by PCR and DNA sequencing analysis. PCR analysis of the genomic DNA was used to assess the deletion of the Cfd gene in the mouse (Figure 1). Genomic DNA was extracted from the tails of mice and analyzed via PCR using primers: Cfd-F1 (5′-AATCTCTCCCCTCTAGAACC-3′); Cfd-R1 (5′-GTCTGTCATGGTGTCTGTTA-3′), Cfd-R2 (5′-GACAGATGATGTGAACCTGT-3′), and Cfd-R3 (5′-CTTCAGTAAACCAGGAAGGG-3′). The reaction program comprised the following steps: 98 °C for 2 min, 40 cycles of 98 °C for 10 s, 58 °C for 10 s and 68 °C for 20 s. The PCR products were cloned into the pCR2.1-TOPO vector (Invitrogen, Carlsbad, CA, USA) and sequenced to determine the exact sequences of both alleles. The DNA sequencing primer is: 5′-GTCAAGCAACCTTCTCTCC-3′ or 5′-GAGGAGGGAGAGATGATG-′. *Cfd*-KO mice were fertile and able to raise pups. We bread all *Cfd*-KO mice for two generations and further bred over several generations.

### 2.3. Reverse Transcription PCR Analysis

Total RNA was extracted using the PureLink RNA Mini Kit (Invitrogen Cat no. 12183018A). First-strand cDNA was synthesized from total RNA (1 μg) with a MultiScribe Reverse Transcriptase Kit (Invitrogen Cat no. 4308228). The Cfd cDNA was amplified with real-time PCR, using the primers Cfd-F (5′-TGCACAGCTCCGTGTACTTC-3′) and Cfd-R (5′-CACCTGCACAGAGTCGTCAT-3′). GAPDH cDNA was amplified with PCR, using the primers GAPDH-F (5′-GGTGAAGGTCGGTGTGAACG-3′) and GAPDH-R (5′-CTCGCTCCTGGAAGATGGTG-3′). The reaction conditions were as follows: 98 °C, 2 min; 40 cycles of 98 °C for 10 s, 60 °C for 10 s, and 68 °C for 30 s. PCR produce size was estimated by electrophoresis on a 2% agarose gel.

### 2.4. Establishment of Murine mADSCs

mADSCs were isolated from the mammary fat pad tissues harvested from the wild-type and Cfd-KO mice, following the protocols previously published. References [11,22] Multiple lines of mADSCs were established from the mammary fat pad tissues of the wild-type and Cfd-KO mice, and early passaged ones were used throughout the experiments. Briefly, adipose tissue was cut into small fragments, put in medium 199 (Thermo Fisher Scientific, Waltham, MA, USA), containing 1 mg/mL collagenase I (Worthington Biochemical, Lakewood, NJ, USA) and 10 unit/mL DNase I (Sigma, St. Louis, MO, USA) and incubated in a shaking water bath at 37 °C for 1 h. After filtration, the cells were collected by centrifugation, and precipitates were resuspended and cultured in Dulbecco’s modified Eagle’s medium/nutrient mixture F12 (DMEM/F12) medium (Gibco, Thermo Fisher Scientific, Waltham, MA, USA) containing 10% fetal bovine serum (FBS), 100 U/mL penicillin and 100 µg/mL streptomycin (Gibco, Thermo Fisher Scientific, Waltham, MA, USA). The cells were maintained in a humidified tissue culture incubator at 37 °C with 5% CO_2_.

### 2.5. Establishment of Breast Cancer Patient-Derived Tumor Xenografts (PDXs)

Human breast cancer PDX was established using surgical specimens of breast cancer patients as previously described [23]. Human breast cancer PDX cells were collected from the dissociated single-cell suspension of the early-passage PDX tumor.

### 2.6. Flow Cytometry

The cells were detached with Accutase (Nacalai, Kyoto, Japan) and blocked with normal mouse IgG (1:100; Wako, Osaka, Japan) and stained with an allophycocyanin (APC)-conjugated anti-mouse CD29 (1:100, clone MMβ1-1, 102215, Biolegend, San Diego, CA, USA), biotin-conjugated anti-mouse CD31 (1:100, clone 390, 13-0311-81, eBioscience, Thermo Fisher Scientific, Waltham, MA, USA), APC-conjugated anti-mouse/human CD44 (1:100, clone IM7 1:20, 103012, Biolegend, San Diego, CA, USA), biotin-conjugated anti-mouse CD45 (1:40, Clone 30-F11, 553078, BD Pharmingen, Franklin Lakes, , NJ, USA), Rat anti-mouse CD49b (1:40, clone DX5, 108901, Biolegend, San Diego, CA, USA), and APC-conjugated anti-mouse CD90.2 (1:100, clone 30-H12 1:20, 105311, Biolegend, San Diego, CA, USA) antibodies, PE-Cy5 streptavidin (1:200, 554062, BD Pharmingen, Franklin Lakes, NJ, USA), goat anti-Rat IgM (Heavy chain) secondary antibody, Alexa Fluor 647 (1:100, A-21248, Invitrogen, Waltham, MA, USA) and PI. Expression levels of cell surface markers were evaluated using a Gallios (Beckman Colter, Brea, CA, USA) or a BD FACSCalibur (BD Bioscience, Franklin Lakes, , NJ, USA) flow cytometer.

### 2.7. Coculture of PDX Cells and mADSCs

Human breast cancer PDX cells (5 × 10^4^ cells/well) and murine mADSCs (1 × 10^4^ cells/well) were well-mixed and plated in a low-attachment 3D culture plate (NanoCulture 96-well plate, low-binding, micro-honeycomb pattern JSR Life Sciences, Tsukuba, Japan). The cells were cultured in DMEM/F12 with 2% FBS, 100 U/mL penicillin, and 100 µg/mL streptomycin. Human HGF (50 ng/mL, 100-39H PeproTech, Cranbury, NJ, USA) was added to the culture medium twice a week. All images of the cultured cells were taken on day 7 after plating using an IX2-SLP microscope (OLYMPUS, Tokyo, Japan). To evaluate sphere-forming ability, the number of spheres larger than 100 µm in diameter was counted.

### 2.8. Adipsin Purification

HEK293 LTV cells were transfected with a pcDNA3.1/V5-HisB plasmid encoding either human or mouse cDNA using Lipofectamine 3000 transfection kit (L3000-015, Invitrogen, Carlsbad, CA, USA). The supernatant media was collected 48 h after transfection and cleared by two successive centrifugation, 2000× *g* at 4 °C for 10 min and 8500× *g* at 4 °C for 10 min. The His-tagged adipsin proteins were purified using a HisPur Ni-NTA purification kit (Thermo Scientific, Waltham, MA, USA) according to the manufacturer’s instructions. Briefly, the HisPur Ni-NTA spin column was equilibrated with an equilibration buffer containing 10 mM imidazole. Approximately 16 mL of clarified medium was applied to a HisPur Ni-NTA spin column and incubated for 30 min at room temperature on a rotator. The medium was discarded by centrifugation at 700× *g* for 2 min. After three times washing using 25 mM imidazole containing wash buffer, the His-tagged protein was eluted using a 3 mL elution buffer containing 250 mM imidazole. In order to exchange the elution buffer and concentrate the product, the eluted fraction was applied to Amicon Ultra-4 centrifugal filter device with 10,000 molecular weight cutoff (MWCO) (No. UFC801008, Merck Millipore, Burlington, MA, USA) according to the manufacturer’s instructions.

### 2.9. Western Blotting

The cells were lysed with a lysis buffer (62.5 mM Tris-HCl (pH 6.8), 10% glycerol, 2.3% SDS, 5% β-mercaptoethanol, 0.2 mg/mL bromophenol blue (BPB)). Samples were separated on SDS-12% polyacrylamide gel electrophoresis and transferred to polyvinylidene difluoride membrane using Trans-Blot Turbo Mini PVDF Transfer system (Bio-Rad, Hercules, CA, USA). After blocking with ImmunoBlock (No. CTKN001, KAC, Kyoto, Japan), filters were incubated with an anti-mouse adipsin (1:400, Clone: AF5430, R&D Systems, Minneapolis, NM, USA) or an HRP conjugated anti-β-actin (1:5000, Clone: AC-15, Sigma) antibody. HRP conjugated donkey anti-sheep immunoglobulin G (1:2000, Clone: 713-035-147, Jackson ImmunoResearch, West Grove, PA, USA) was then added, and the bands were detected using the Chemi-Lumi One Ultra (Nacalai, Kyoto, Japan) and the Fusion Solo S (Vilber, Collégien, France). Intensities of protein bands were quantitated using the ImageJ Gel Analysis program. All the whole western blot figures can be found in the Supplementary Materials.

### 2.10. Cytokine Array

mADSCs were 3D-cultured for 3 days. The culture supernatants were collected by centrifugation at 300× *g* for 10 min at 4 °C. The expression profile of the 111 murine cytokines was analyzed using Proteome Profiler Mouse XL Cytokine Array Kit (R&D Systems, Minneapolis, NM, USA), according to the manufacture’s instruction. Images of the membrane were taken using the Fusion Solo S (Vilber, Collégien, France).

### 2.11. Xenotransplantation Assay

Breast cancer PDX cells and mADSCs were mixed, suspended in Matrigel (Corning, Glendale, AZ, USA), and then injected into the mammary fat pad region of female BRJ mice [24], NOD/SCID mice (CLEA, Osaka, Japan), or NSG mice (Charles River, Wilmington, MA, USA). Four hundred thousand PDX cells and 8 × 10^4^ mADSCs were cocultured to form tumorspheres for three days with or without human HGF (50 ng/mL, 100-39H, PeproTech, Cranbury, NJ, USA) in a low-attachment 3D culture plate (NanoCulture 96-well plate, low-binding, micro-honeycomb pattern JSR Life Sciences, Tsukuba, Japan); and then tumorspheres were detached from the plate and injected into mammary fat pad region of female mice. After transplantation, HGF (2.5 ng/injection) was subcutaneously injected twice a week. Tumor sizes were measured twice a week and tumor volumes (mm^3^) were estimated using the formula: volume = ab^2^/2 [a length; b, width (mm)] [4].

### 2.12. Statistical Analysis

Data are presented as means ± standard deviation (SD). Comparisons between continuous data normally distributed with equal variance or unequal variances between groups were performed using unpaired two-tailed Student’s *t*-tests. Sample sizes, statistical tests, and *p*-values are indicated in the figures or figure legends. All *p*-values were two-sided, and *p*-values < 0.05 were deemed statistically significant. Asterisks denote *p*-value significance.

## 3. Results

### 3.1. Generation of Adipsin-KO Mouse and Mammary ADSCs

**Figure 2:**
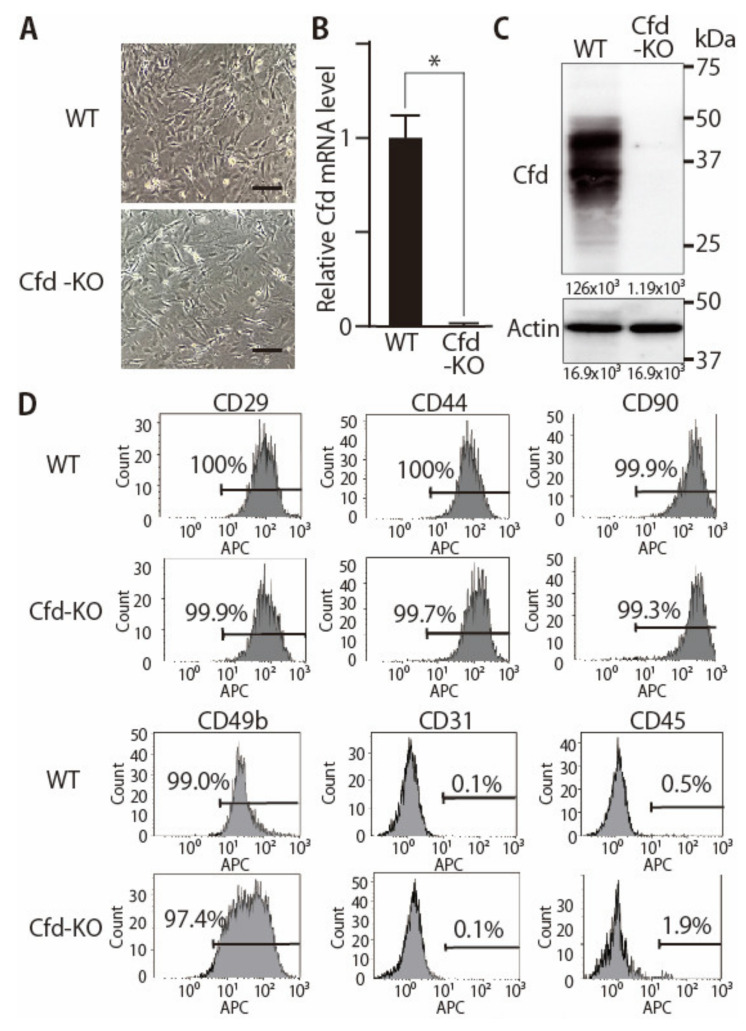
Establishment of Cfd-KO mADSCs. (**A**) Microscopic appearance of WT and Cfd-KO mADSCs. Scale bar: 50 μm. (**B**) The expression level of *Cfd* mRNA in mADSCs. *Cfd* mRNA was undetectable in Cfd KO mADSCs. *GAPDH* was used as a control. * *p* < 0.05. (**C**) The expression level of Cfd protein in differentiated mADSCs. Murine Cfd protein is composed of a mixture of glycosylated isoforms with two prominent bands observed between 30 and 50 kDa [27]. Cfd protein was undetectable in the cell lysate of Cfd-KO mADSC. Actin was used as a control. (**D**) Expression levels of cell surface markers in WT and KO mADSCs. Expression levels were analyzed using a flow cytometer. The percentage of cells positive for cell surface marker expression is presented.

Adipocyte is a component of the tumor microenvironment in breast cancers. We have reported that adipokine adipsin (CFD) is a mediator of adipocyte-cancer cell interaction in human breast cancers [11]. Based on these findings, we generated Cfd-KO mice using the CRISPR/Cas9 gene editing system (Figure 1A). We designed two sgRNAs targeted on the upstream and downstream of the full coding sequence of the mouse *Cfd* gene. Deletion of *Cfd* in the mouse was confirmed using the PCR analysis and sequencing of the genomic DNA of the tail (Figure 1). Wild-type (WT) and Cfd-KO mammary ADSCs (mADSCs) were established from the mammary fat pad of the WT and Cfd-KO mouse, respectively (Figure 2A). *Cfd* mRNA and protein were detectable in WT ADSCs, but undetectable in Cfd-KO mADSCs (Figure 2B,C). Adipsin (Cfd) is a serine protease that is involved in the conversion of complement C3 to the small fragments C3a and C3b [25]. Consistent with the previous findings [11,26], the murine mADSCs were characterized by highly ubiquitous expression of the cell surface markers CD29, CD44, CD49b, and CD90, and lacked the expression of endothelial and hematopoietic lineage markers CD31 and CD45 (Figure 2D). The distribution patterns of CD29, CD44, and CD90 in the histograms were similar, but that of CD49b was broader in Cfd-KO mADSCs than in WT mADSCs (Figure 2D).

### 3.2. Adipokine Cfd-Dependent Tumorsphere Formation by Breast Cancer PDX Cells

**Figure 3:**
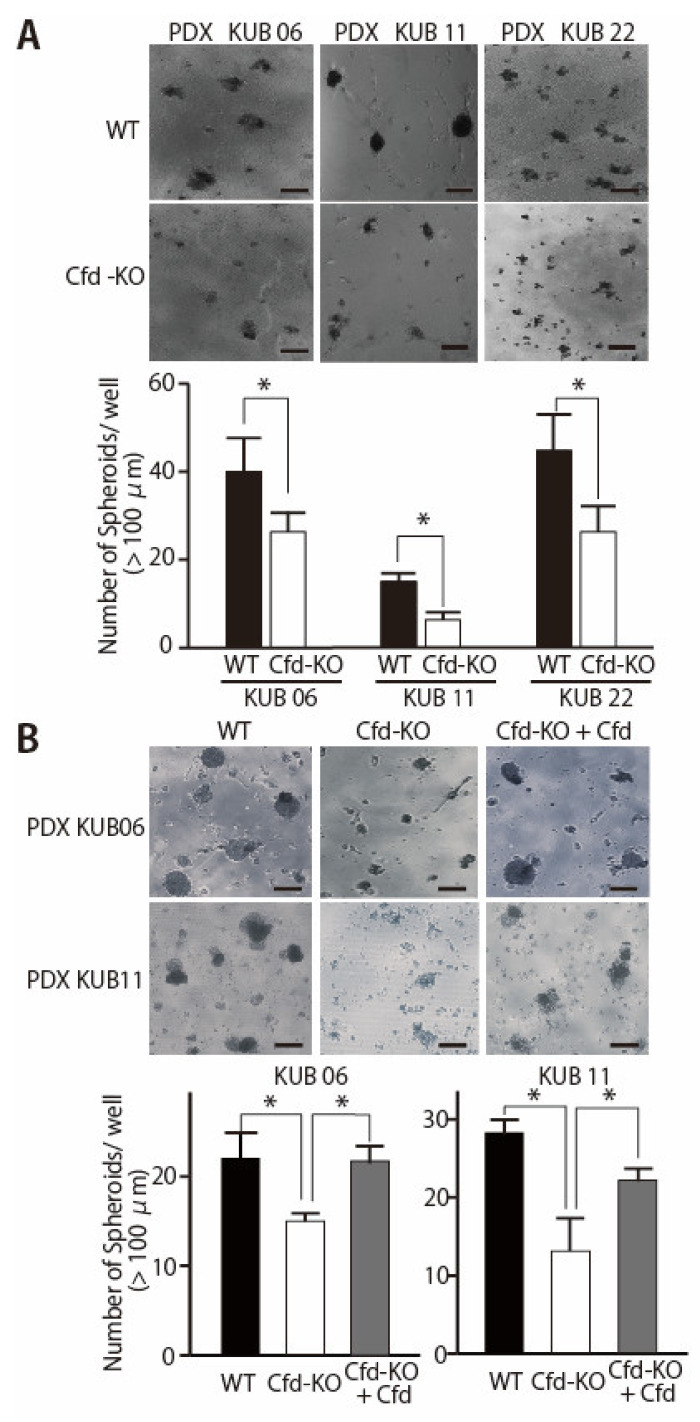
Cfd-KO in mADSCs suppressed the tumorsphere formation of cocultured breast cancer PDX cells. (**A**) Representative images of the tumorspheres formed by coculture of breast cancer PDX cells (PDX KUB06, KUB11, or KUB22) with WT or Cfd-KO mADSCs. Scale bar, 100 μm. The bar graph shows the number of tumorspheres (>100 μm in diameter) formed by PDX cells cocultured with mADSCs. * *p* < 0.05. (**B**) Cfd rescued the reduced ability of Cfd-KO mADSCs to induce sphere formation of breast cancer PDX cells. Representative images of the tumorspheres formed by coculture of breast cancer PDX cells (PDX KUB06, or KUB11) with Cfd-KO mADSCs with or without Cfd (9.5 μg/mL) in the culture medium were presented. Scale bar, 100 μm. The bar graph shows the number of tumorspheres (>100 μm in diameter) formed by PDX cells cocultured with Cfd-KO mADSCs. * *p* < 0.05.

To evaluate the ability of mADSCs to enhance the sphere formation abilities of breast cancer PDX cells, human breast cancer PDX cells (KUB06, 5 × 10^4^ cells/well) were cocultured with mADSCs (1 × 10^4^ cells/well) using the 3D-coculture system that we had previously reported [11]. Coculture with WT mADSCs significantly increased the number of spheres formed by PDX cells, while a significant decrease was observed when cocultured with Cfd-KO mADSCs (Figure 3A). Essentially identical findings were observed when Cfd-KO mADSCs were cocultured with other breast cancer PDXs derived from distinct breast tumors (Figure 3A). The sizes of smaller tumorspheres did not increase even after the longer incubation. The addition of purified Cfd (9.5 μg/mL) in the culture medium significantly increased the number of tumorspheres formed by PDX cells cocultured with Cfd-KO mADSCs, further confirming that tumorsphere formation was at least partly dependent on Cfd (Figure 3B). These results suggest that Cfd secreted from mADSCs enhances the CSC properties of breast cancer PDX cells.

### 3.3. HGF Alleviated the Reduced Ability of Cfd-KO mADSCs to Promote Tumorsphere Formation

**Figure 4:**
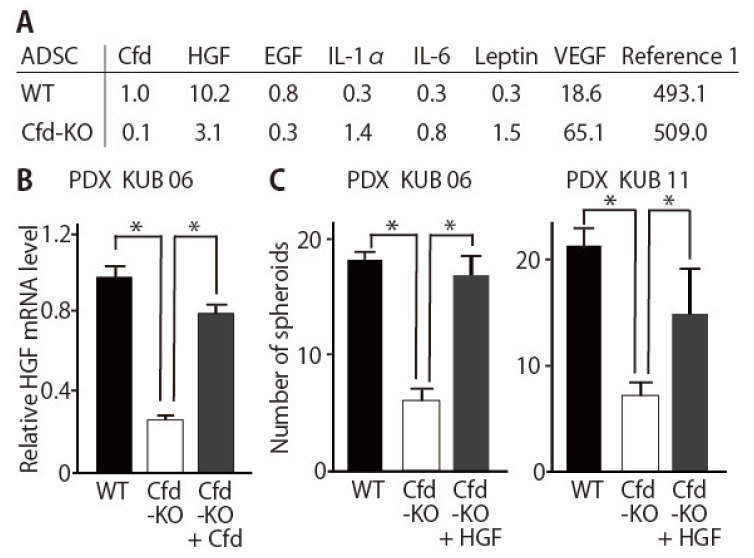
HGF rescues the reduction of tumorsphere formation by Cfd-KO mADSCs. (**A**) Profiling of adipokines secreted from WT- and Cfd-KO mADSCs. Culture medium of mADSCs was analyzed using cytokine arrays. The intensity of the spot for Cfd in WT mADSCs was set at 1.0. (**B**) Reduction of the HGF mRNA levels in the Cfd-KO mADSCs. The addition of Cfd in the culture medium (Cfd, 9.5 μg/mL) upregulated the expression level of Cfd mRNA in the Cfd-KO mADSCs. * *p* < 0.05. (**C**) HGF rescued the reduced ability of Cfd-KO ADSCs to induce sphere formation of breast cancer PDX cells. HGF (50 ng/mL) was added to the culture medium. The number of PDX tumorspheres (>100 μm in diameter) were presented. Scale bar, 100 μm. * *p* < 0.05.

Cfd has multiple roles other than the cleavage of C3 to produce C3a and C3b in the alternative pathway of the complement system [13]. Profiling of adipokines secreted from Cfd-KO mADSCs revealed that secretion of multiple adipokines, including HGF and EGF, were modulated by Cfd-KO (Figure 4A, Supplementary Table S1). The expression level of HGF in Cfd-KO mADSCs was increased when Cfd was added to the culture medium (Figure 4B). The addition of HGF in the culture medium increased the number of tumorspheres formed by breast cancer PDX cells cocultured with Cfd-KO mADSCs (Figure 4C). The mRNA expression levels of CSC-related genes CD44, ZEB1, and SNAI1, and the cell surface expression level of CD44 were significantly lower when breast cancer PDX cells were cocultured with Cfd-KO mADSCs than when cocultured with WT mADSCs; and were recovered when HGF was added to the culture medium (Supplementary Figure S1). In contrast, the addition of EGF in the culture medium increased the size of tumorspheres formed by breast cancer PDX cells cocultured with Cfd-KO mADSCs but failed to rescue the number of tumorspheres formed by cocultured PDX cells (Supplementary Figure S2). Furthermore, a combination of HGF and EGF did not show a synergistic effect to increase the number of tumorspheres (Supplementary Figure S3). These results suggest that HGF is a downstream effector of Cfd in mADSCs that promotes the sphere formation of breast cancer PDX cells in vitro.

### 3.4. HGF Alleviated the Reduced Effect of Cfd-KO mADSCs on Tumor Formation

**Figure 5:**
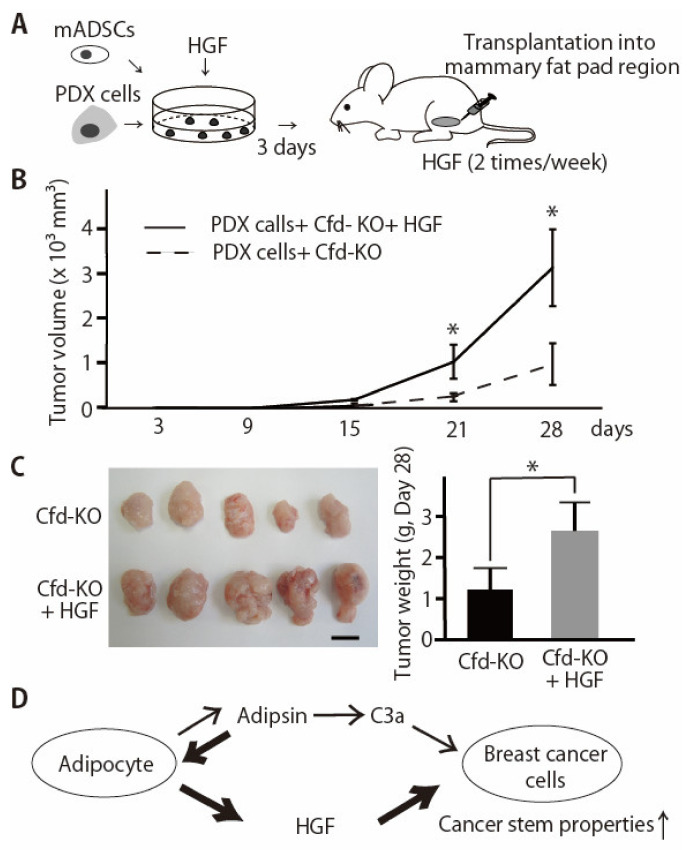
HGF enhanced the tumor formation by breast cancer PDX cells co-injected with Cfd-KO mADSCs. (**A**) Schematic presentation of experimental procedures. Breast cancer PDX cells and mADSCs were cocultured for three days with or without HGF (50 ng/mL) and xenotransplanted to the mammary fat pad regions of the immunodeficient mice. (**B**) HGF enhanced the tumor formation by breast cancer PDX cells co-injected with Cfd-KO mADSCs. Four hundred thousand breast cancer PDX cells (KUB06) and 8 × 10^4^ Cfd-KO mADSCs were cocultured with or without HGF (50 ng/mL) for 3 days in vitro and xenotransplanted in the mammary fat pad regions of the immunodeficient mice (*n* = 5). HGF or control PBS was subcutaneously injected twice a week. * *p* < 0.05. (**C**) The appearance of the xenograft tumor. Scale bar, 10 mm. The bar graph shows the weight of tumors at day 28. * *p* < 0.05. (**D**) Schematic illustration of the molecular functions of adipsin (Cfd) in the adipocyte-breast cancer cell interaction. 1. Anaphylatoxin C3a, the product of Cfd in the alternative complement pathway, functions as an activator of the CSC properties of breast cancer PDX cells [11]. 2. HGF is a downstream effector of Cfd secreted from mADSCs and enhances the CSC properties of breast cancer PDX cells.

CSC properties are associated with tumor initiation and progression. We have previously shown that CFD-knockdown in mADSCs using sh-CFD expression lentivirus significantly reduced the ability of mADSCs to enhance tumor growth of breast cancer PDX cells in vivo [11]. Because HGF, but not EGF, significantly alleviated the suppressive effect of Cfd-KO mADSCs on tumorsphere formation in vitro (Figure 4, Supplementary Figure S2), we evaluated the effect of HGF to alleviate the suppressive effect of Cfd-KO mADSCs on tumor formation in vivo. Tumorspheres were formed by coculturing PDX cells and mADSCs for three days with or without HGF and then xenotransplanted in the immunodeficient mice (Figure 5A). HGF-treated PDX tumors grew significantly faster than control PDX tumors (Figure 5B). The tumor weight of HGF-treated PDX tumors was significantly higher than untreated PDX tumors (Figure 5C). These results suggest that HGF is a downstream effector of Cfd for the enhancement of CSC properties and tumor formation in breast cancers (Figure 5D).

## 4. Discussion

Growing evidence proposed an adipose-epithelial cell interaction as an active player in the tumor microenvironment [28]. In fact, obesity, which is characterized by the excess of adipose tissue, is associated with an increased risk of multiple cancers, including postmenopausal breast, endometrial, and colorectal cancers [29]. Various mechanisms have been proposed to explain how mature adipocytes alter breast cancer cell behavior, including secretion of adipokines, remodeling of the extracellular matrix, enhanced inflammation, and metabolic changes [30,31,32,33]. Furthermore, bone marrow adipocytes are responsible for bone metastasis [34,35]. The best described of these involves secreted factors (adipokines), such as leptin, adiponectin, interleukin-6, and insulin-like growth factor 1. We have previously shown that adipokine adipsin (CFD) secreted predominantly from mADSCs enhances the proliferation and CSC properties of breast cancer PDX cells [11].

While most of the complement factors are produced in the liver, CFD is mostly produced in the adipocytes. The reason why adipose tissue is involved in the activation of innate immunity has not been fully elucidated. In this study, we established Cfd-KO mice and established mADSCs from them (Figure 1 and Figure 2). We then showed that Cfd regulated the expression profiles of adipokines, including HGF and EGF (Figure 5D, Supplementary Table S1). These results indicate that in addition to its role in innate immunity, Cfd functions as an autocrine regulator of adipocytes. Furthermore, our results showed that HGF was a downstream effector of Cfd and more effectively enhanced the CSC properties of breast cancer PDX cells than EGF (Figure 4, Supplementary Figure S2).

HGF is a growth factor that binds to MET receptor, and human and murine HGF proteins are more than 90% identical and 96% similar. HGF/MET signaling promotes CSC properties by inducing YAP nuclear translocation and HIF-1α stabilization in pancreas cancer [36]. Among the growth factors secreted from mesenchymal cells, HGF, IL-6, VEGF, IL-8, IL-23 are the factors that significantly enhance the CSC properties [37]. Specific roles of HGF in the maintenance of CSCs are also reported in glioblastoma, colorectal, and prostate cancers [38,39,40,41]. In contrast, although VEGF is an enhancer of CSC properties [42], upregulation of VEGF by Cfd-KO did not appear to have affected the tumorsphere formation (Figure 4A).In addition, at least partly consistent with previous observation [37], EGF did not promote the CSC properties (Supplementary Figures S2 and S3). Further studies are required to clarify relatively specific roles of HGF in the adipocyte-CSC interactions and stem cell niche. As presented in colorectal CSCs [43], it is highly reasonable to speculate that breast CSCs acquire niche independence and become more independent from CFD, HGF, and/or other factors during metastatic progression. Indeed, activation of c-MET signaling in breast cancer cells promotes metastasis of breast cancer cells and secretion of HGF from tumor-associated astrocytes in the brain [44].

We have previously reported that shRNA-mediated knockdown of CFD in human mammary ADSCs suppresses their ability to support tumorsphere formation of PDX cells [11]. However, probably because of the residual amount of CFD produced by sh-CFD ADSCs, the suppressive effect is weaker than those achieved by the specific inhibitor of CFD-C3a signaling, SB290157. In this study, using Cfd-KO mADSCs, we showed that the sphere formation ability of PDX cells was clearly and significantly suppressed by Cfd-KO in mADSCs. The sequences of CFD and Cfd are 66% identical and all three residues critical for enzymatic activity are conserved between them [45] (Supplementary Figure S4). These observations further support our notion that adipsin (CFD) is an active player of adipocyte-cancer cell interactions. Because the addition of Cfd rescued the reduction of tumorsphere formation by PDX cells cocultured with mADSCs, murine Cfd was functional in this culture system.

## 5. Conclusions

Adipsin (Cfd)-KO mADSCs significantly reduced their ability to enhance tumorsphere formation that reflected CSC properties of breast cancer PDX cells. We then found that the secretion of HGF from mADSCs was Cfd-dependent, and HGF effectively alleviated the suppressive effect of Cfd-KO on tumorsphere formation. These results suggest that adipsin and its downstream effector HGF are active players of adipocyte-cancer cell interactions.

## Data Availability

The data of the cytokine arrays in this study are available as Supplementary Materials.

## Supplementary Materials

The following are available online at https://www.mdpi.com/article/10.3390/cancers13164238/s1, Table S1: Adipokine expression profiles of WT and Cfd-KO mADSCs, Figure S1: The effect of HGF on the expression levels of CSC markers in breast cancer PDX tumorspheres, Figure S2: EGF failed to rescue the reduction of tumorsphere formation by Cfd-KO mADSCs, Figure S3: No synergistic effect of the combination of EGF and HGF on the reduction of tumorsphere formation by Cfd-KO in mADSCs: Figure S4: Protein sequences of human CFD and murine Cfd.
