# Supplementary material for: Adipsin-Dependent Secretion of Hepatocyte Growth Factor Regulates the Adipocyte-Cancer Stem Cell Interaction
Source: Cancers (Basel). 2021 Aug 23;13(16):4238. doi: 10.3390/cancers13164238 (PMC8393397; doi:10.3390/cancers13164238)
Supplement: Supplementary file 1 [file cancers-13-04238-s001.zip › Supplementary legends for Figures and Table.pdf]

Supplementary Appendix

Adipsin-dependent secretion of hepatocyte growth factor regulates the adipocyte-cancer stem cell interaction

Supplementary Table and Figures

Supplementary Table S1. Adipokine expression profiles of WT and Cfd-KO mADSCs.

Supplementary Figure S1. The effect of HGF on the expression levels of CSC markers in breast cancer PDX tumorspheres. **A.** The expression levels of CSC markers. Breast cancer PDX cells (PDX KUB06) were co-cultured with WT or Cfd-KO mADSCs for 3 days. HGF (50 ng/ml) was added to the culture medium. The expression levels of CSC markers were measured by semi-quantitative PCR. $*p < 0.05$. **B.** Reduction of CD44 expression in breast cancer PDX cells co-cultured with Cfd-KO mADSCs. The expression level of CD44 in KUB06 PDX cells was analyzed using a flow cytometer.

Supplementary Figure S2. EGF failed to rescue the reduction of tumor sphere formation by Cfd-KO mADSCs. EGF did not rescue the reduced ability of Cfd-KO ADSCs to induce sphere formation of breast cancer PDX cells. EGF (100 ng/ml) was added in the culture medium. Representative images of the tumorspheres formed and number of PDX tumor spheres (> 100 µm in diameter) were presented. Scale bar, 100 µm. $*p < 0.05$; NS, not significant.

Supplementary Figure S3. No synergistic effect of the combination of EGF and HGF on the reduction of tumorsphere formation by Cfd-KO in mADSCs. Combined addition of both EGF and HGF did not show the additive effect on the reduced ability of Cfd-KO ADSCs to induce sphere formation of breast cancer PDX cells. Both EGF (100 ng/ml) and HGF (50 ng/ml) were added in the culture medium. Representative images of the tumorspheres formed and number of PDX tumor spheres (> 100 µm in diameter) were presented. Scale bar, 100 µm. $*p < 0.05$; NS, not significant.

Supplementary Figure S4. Protein sequences of human CFD and murine Cfd. Three residues critical for the enzymatic activity to produce C3a are highlighted in purple (ref: Structure of Human Factor D A Complement System Protein at 2.0 A Resolution. Sthanam V. L. Narayana, J. Mol. Biol. (1994) 235, 695-708). The residues identical between CFD and Cfd are highlighted in black, and those with similar character are highlighted in grey. CFD, 253 amino acids; Cfd, 259 amino acids. The sequence of human CFD is 66% identical and 80% similar to murine Cfd.
