# Supplementary material for: Adipsin-Dependent Secretion of Hepatocyte Growth Factor Regulates the Adipocyte-Cancer Stem Cell Interaction
Source: Cancers (Basel). 2021 Aug 23;13(16):4238. doi: 10.3390/cancers13164238 (PMC8393397; doi:10.3390/cancers13164238)
Supplement: Supplementary file 1 [file cancers-13-04238-s001.zip › Supplementary Figure S4.pdf]

```
Human CFD      1  MHSWERLAVLVLLGAAACAAPPRGRILGGREAEAHARPYMASVQLNGAHLCGGVLVAEQW
Murine Cfd     1  MHSSVYFVALVILGAAVCAAQPRGRILGGQEAAAHARPYMASVQVNGTHVCGGTLLDEQW

Human CFD     61  VLSAAHCLEDAAD-GKVQVLLGAHSLSQPEPSKRLYDVLRAVPHPDSQPDTIDHDLLLLQ
Murine Cfd    61  VLSAAHCMDGVTDDDSVQVLLGAHSLSAPEPYKRWYDVQSVVPHPGSRPDSLEDDLILFK

Human CFD    120  LSEKATLGPAVRPLPWQRVDRDVAPGTLCDVAGWGIVNHAGRRPDSLQHVLLPVLDRATC
Murine Cfd   121  LSQNASLGPHVRPLPLQYEDKEVEPGTLCDVAGWGVVTHAGRRPDVLHQLRVSIMNRTTC

Human CFD    180  NRRTHHDGAITERLMCAESNRRDSCKGDSGGPLVCGGVLEGVVTSGSRVCGNRKKPGIYT
Murine Cfd   181  NLRTYHDGVVTINMMCAESNRRDTCRGDSGSPLVCGDAVEGVVTWGSRVCGNGKKPGVYT

Human CFD    240  RVASYAAWIDSVLA-----
Murine Cfd   241  RVSSYRMWIENITNGNMTS
```

Mizuno M et al. Supplementary Figure 4
