# Supplementary figures and images for: Adipsin-Dependent Secretion of Hepatocyte Growth Factor Regulates the Adipocyte-Cancer Stem Cell Interaction
Source: Cancers (Basel). 2021 Aug 23;13(16):4238. doi: 10.3390/cancers13164238 (PMC8393397; doi:10.3390/cancers13164238)

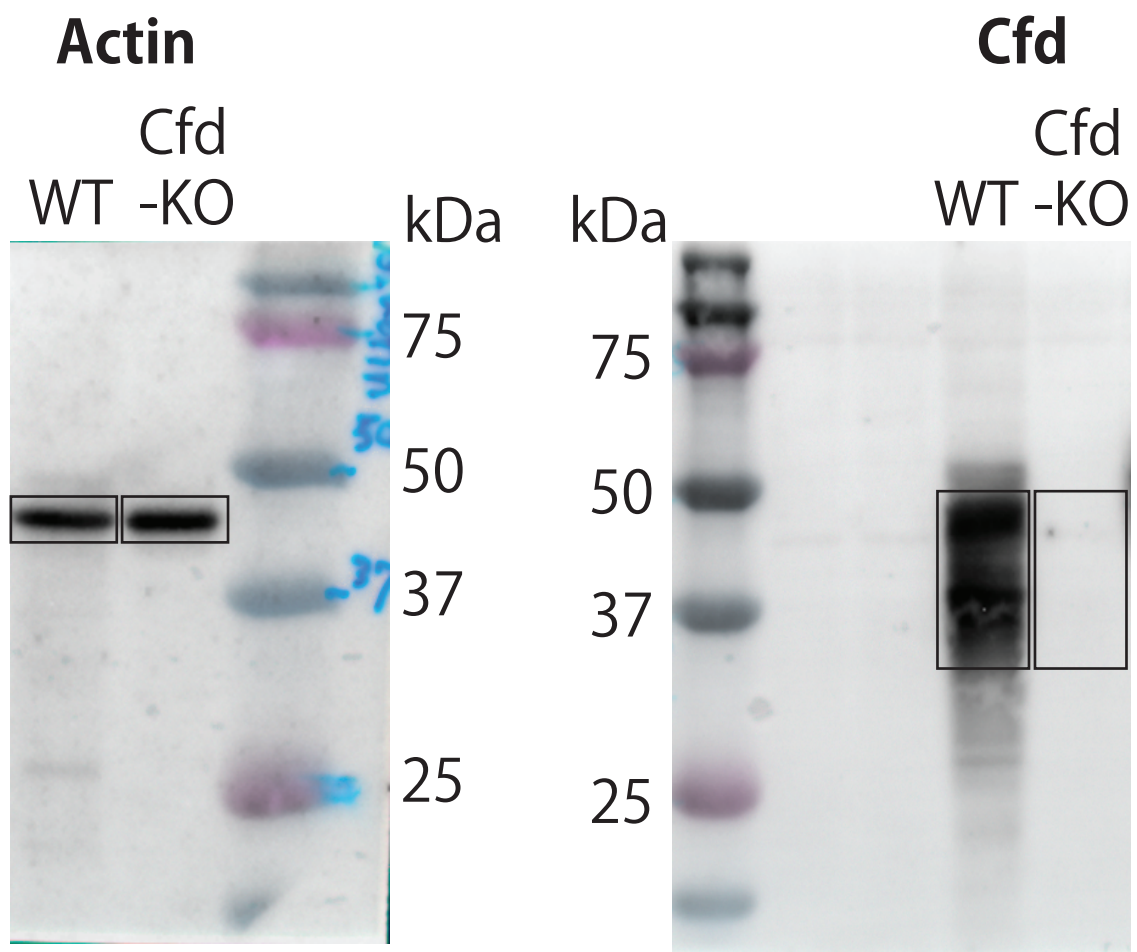

Original blot for Figure 2C

Mizuno M et al.

Supplement: Supplementary file 1 [file cancers-13-04238-s001.zip › Original blot Figure 2C.pdf]

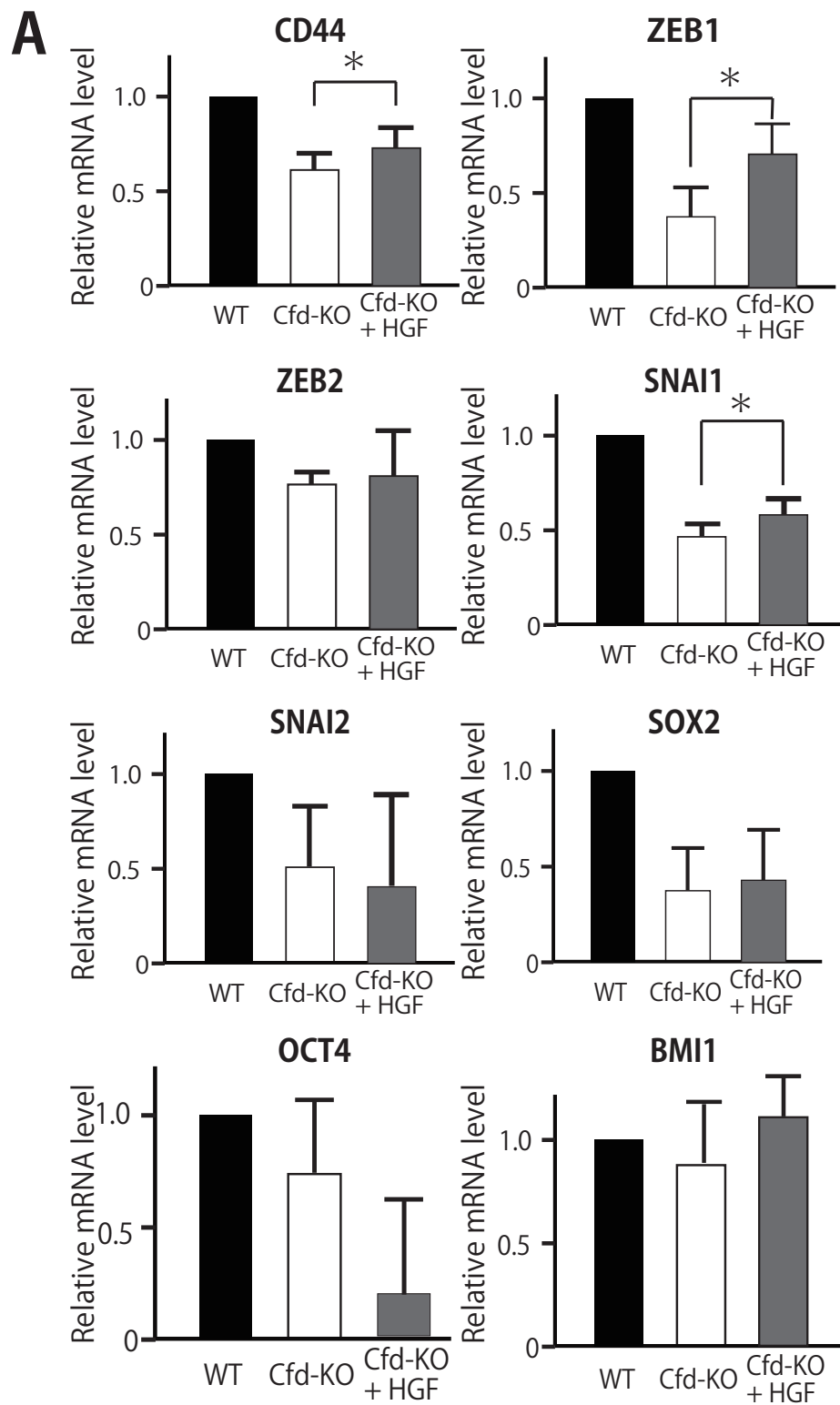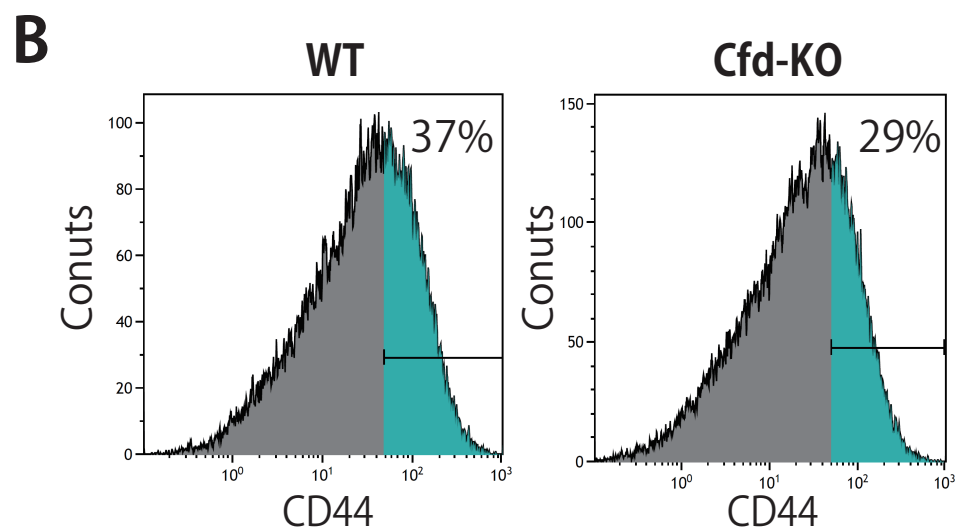

Supplement: Supplementary file 1 [file cancers-13-04238-s001.zip › Supplementary Figure S1.pdf]

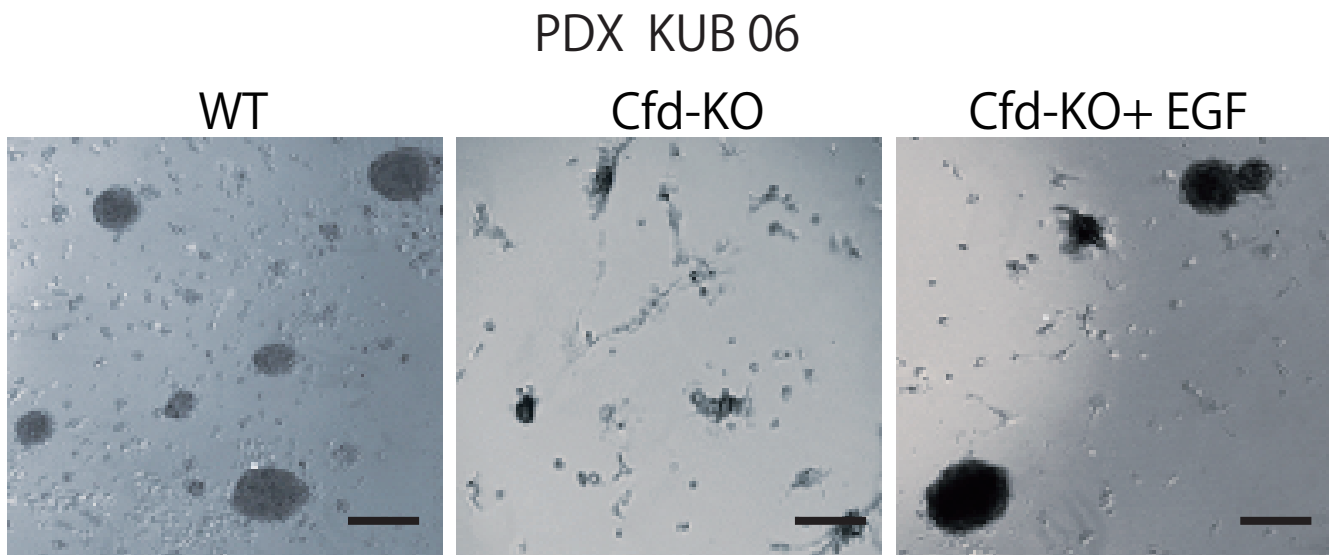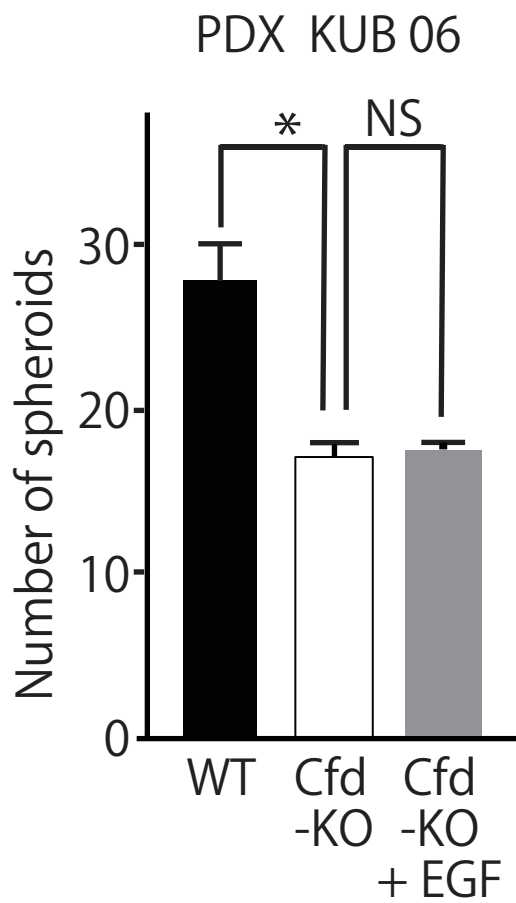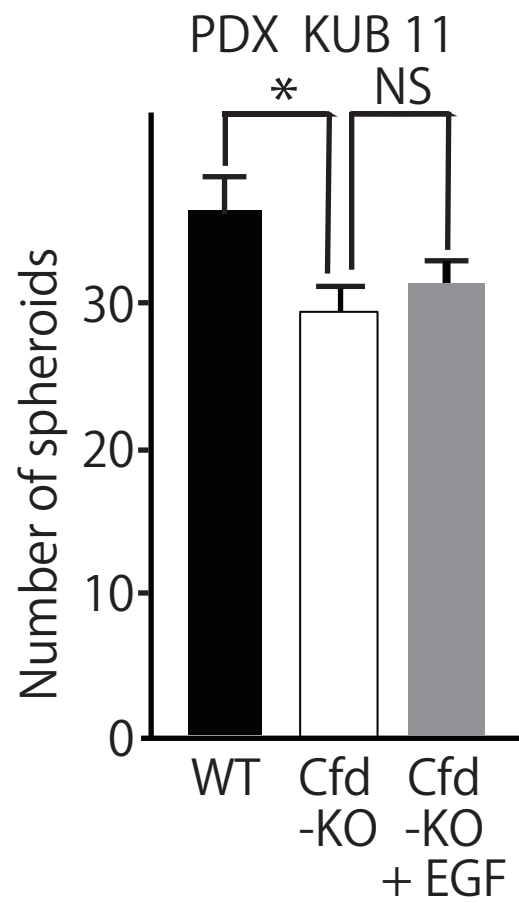

Supplement: Supplementary file 1 [file cancers-13-04238-s001.zip › Supplementary Figure S2.pdf]

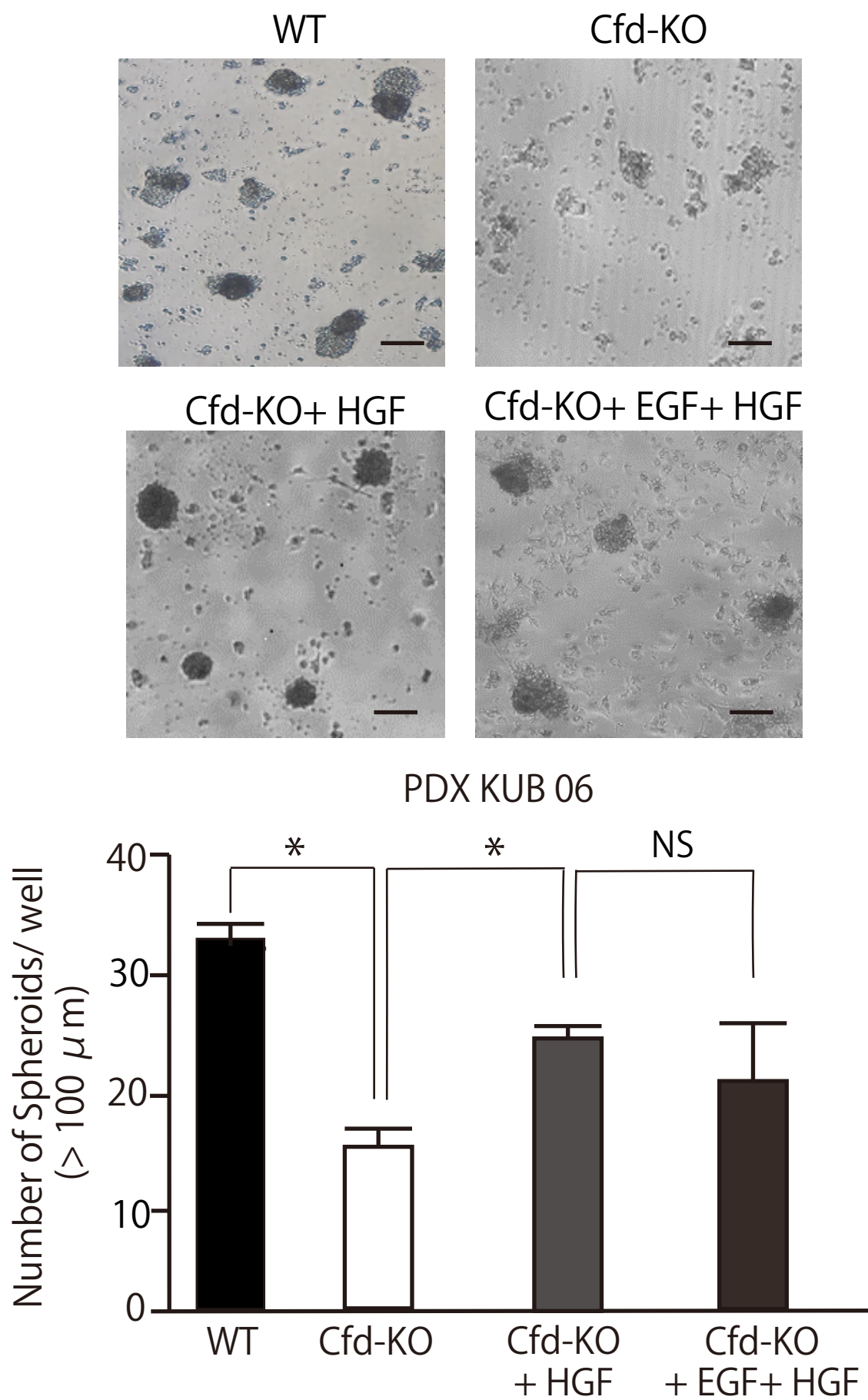

Supplement: Supplementary file 1 [file cancers-13-04238-s001.zip › Supplementary Figure S3.pdf]
